# Preparation and Biological Evaluation of Two Novel Platinum(II) Complexes Based on the Ligands of Dipicolyamine Bisphosphonate Esters

**DOI:** 10.3390/molecules21030255

**Published:** 2016-02-24

**Authors:** Ling Qiu, Hong Liu, Ke Li, Gaochao Lv, Hui Yang, Xiaofeng Qin, Jianguo Lin

**Affiliations:** Jiangsu Key Laboratory of Molecular Nuclear Medicine, Key Laboratory of Nuclear Medicine, Ministry of Health, Jiangsu Institute of Nuclear Medicine, Wuxi 214063, China; qiuling@jsinm.org (L.Q.); lhgnsfxy@hotmail.com (H.L.); like@jsinm.org (K.L.); lvgaochao@jsinm.org (G.L.); nmuyanghui@sina.com (H.Y.); qinxiaofeng@jsinm.org (X.Q.)

**Keywords:** dipicolyamine bisphosphonate ester, platinum complex, hydroxyapatite affinity, antitumor activity, action mechanism

## Abstract

Two new platinum(II)-based complexes bearing a bone-targeting group were synthesized and characterized. They both have excellent affinity for hydroxyapatite (HA), which is abundant in human bone tissues. Their antitumor activities against five human cancer cell lines (U2OS, A549, HCT116, MDA-MB-231 and HepG2) were evaluated and compared with cisplatin (CDDP). Though the antitumor efficacies of new complexes are lower than that of CDDP, they show higher selectivity against the HepG2 hepatoma cell line than the L02 normal liver cell line. Morphology studies exhibited typical characteristics of cell apoptosis and the cell cycle distribution analysis indicated that the complexes can inhibit cancer cells by inducing cell cycle arrest at the G2/M phase, a similar mechanism of action to CDDP.

## 1. Introduction

It is well known that the rate of incidence of cancer is increasing rapidly on a yearly basis around the world, which presents a serious threat to our health and life. One of the most widely-used drugs in the clinic are the platinum-based complexes cisplatin and carboplatin, which are extensively employed in the treatment of ovarian, lung, head, and neck cancers, and oxaliplatin, which is widely used in treating colorectal cancer [1]. The antitumor mechanism of these compounds arises from their abilities to form intra- and inter-strand cross-links on DNA via coordination of the N7 atoms of purine bases to the platinum center. These cross-links can inhibit the activities of DNA and RNA polymerases, which can also initiate a signaling cascade that leads to cell death. However, the use of platinum-based antitumor drugs is restricted by their serious side effects and drug resistance [2]. A huge number of works has been done to try to overcome these drawbacks. One strategy to improve their therapeutic efficacy and decrease their side effects is to enhance their tumor-targeting selectivity [3,4].

Among all kinds of cancer, osteosarcoma is one of the most painful malignant tumors and it is hard to treat due to the particular physiological and biochemical processes of bone tissue. It was hypothesized that anticancer drugs with bone-targeting groups could enhance the anticancer effect against osteosarcoma and decrease the side effects. Bisphosphonates (BPs) have been widely used as therapeutic agents for treating several bone-related diseases, such as metabolic bone diseases and bone tumors [5,6]. BPs have high affinity for bone minerals, which makes bisphosphonate a suitable bone-targeting group to improve a drug’s selectivity towards bone tumors. Recently, several anticancer platinum complexes with bisphosphonate groups have been explored. Keppler synthesized platinum complexes bearing a phosphoric acid as the bone-targeting group in the early 1990s [7]. Natile *et al*. have synthesized novel dinuclear platinum complexes containing bisphosphonates, which showed high affinity for bone tumors or metastases [8]. They further designed and synthesized a series of dinuclear platinum complexes containing the bisphosphonate group [9,10,11,12,13]. The biological property evaluation results showed that these platinum complexes have great binding affinity for hydroxyapatite (HA) and have the ability to inhibit the proliferation of human osteosarcoma (U2OS) and lung adenocarcinoma (A549) cell lines. Xue *et al.* [14] designed and synthesized a series of novel platinum complexes which linked BPs in non-leaving groups to stop the bisphosphonate moieties from being released before reaching the bone tissues. The platinum complexes showed considerable cytotoxicity against human osteosarcoma (MG-63) and ovarian (COC1) cancer cell lines, and they induced cell death by a different mechanism than cisplatin [14]. In order to increase the solubility and enhance the transport through cellular membranes, phosphonate esters were used as functional ligands to design platinum complexes [15]. Since phosphonate esters can be hydrolyzed under biological conditions, the complexes based on phosphonate esters still maintain the targeting function. Therefore, this strategy will be conducive to discovering novel bone-targeting platinum drugs based on the ligand containing phosphonate esters.

**Figure 1:**
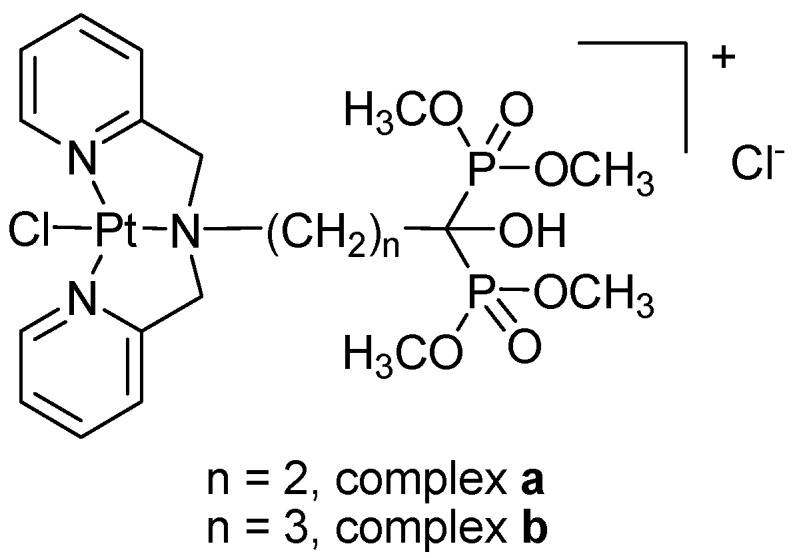
Chemical structures of the designed platinum complexes.

In this study, two novel platinum complexes based on the dipicolyamine bisphosphonate ester moiety were designed and synthesized, [Pt(DPE)Cl]Cl (**a**) and [Pt(DAE)Cl]Cl (**b**) (DPE = dipicolylaminepamidronate ester, DAE = dipicolylaminealendronate ester, Figure 1). The non-leaving functional moieties contain the *N*-heterocyclic ring as coordination group to the platinum center and the phosphonate ester as targeting group for specific tissues. Their biological activities were systematically evaluated *in vitro*, such as their cytotoxic effect on human cancer cell lines and human normal cell lines as well as bone-targeting ability. Moreover, their mechanism of action was also studied by means of nuclear staining, flow cytometry and circular dichroism (CD) spectroscopy techniques.

## 2. Results and Discussion

### 2.1. Preparation and Characterization

The ligands dipicolylaminepamidronate ester (DPE) and dipicolylaminealendronate ester (DAE) were prepared according to the previously reported method [16,17]. Briefly, dipicolylamine-pamidronate was first obtained in high yield by mixing two equivalents of 2-(chloromethyl)pyridine with the corresponding aminobisphosphonates in a aqueous solution under pH = 12. Then, the dipicolylaminepamidronate ester was obtained in moderate yield by treating the dipicolylamine-pamidronates with excess trimethylsilyldiazomethane in the methanol solution. The two complexes [Pt(DPE)Cl]Cl (**a**) and [Pt(DAE)Cl]Cl (**b**) were respectively synthesized in a satisfactory yield by mixing equimolar amounts of the ligand and *cis*-Pt(DMSO)_2_Cl_2_ [18] in methanol solution. The elemental analyses and mass spectrometry data of the resulting complexes are in line with the formation of mononuclear platinum(II) complexes. The ^1^H-, ^13^C-, and ^31^P-NMR spectra (Figures S3–S14 of Supplementary Materials) match well with the structures of complexes, and they are similar to those of corresponding ligands. There is no obvious downfield shift of the phosphorus nuclei in the NMR spectra, indicating that the phosphonate groups do not coordinate to the Pt center. The Pt(II) center is coordinated by three N atoms of bis(2-pyridylmethyl)amine (BPA) to form the mononuclear platinum center, and this chelating mode has been demonstrated in the reported crystal structures of platinum complexes with BPA chelating agents [19,20].

### 2.2. Lipid-Water Partition Coefficient

The lipid-water partition coefficient (log *P*) of complexes **a** and **b** was determined to be −1.14 and −1.09, respectively, which suggested that both complexes are hydrophilic and it is conducive to bone-targeting ability. The lipid-water partition can influence the uptake of drug through some biological processes, such as transport through membranes [21]. The hydrophilicity of platinum complexes might make it more difficult to pass through the cell membrane, and hence the drug uptake is relatively low. This may lead to a relatively lower antitumor effect than those of the platinum-based anticancer agents used in the clinic.

### 2.3. Bone Binding

**Figure 2:**
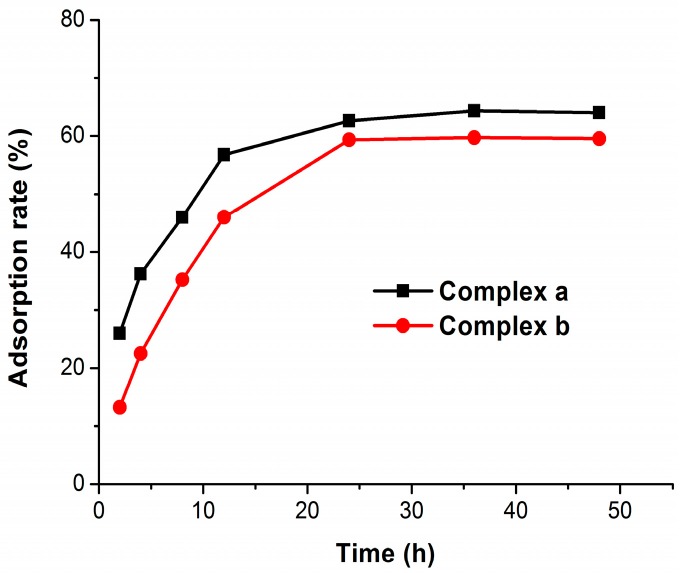
Adsorption curves of complexes **a** and **b** onto HA.

Active targeting of therapeutic agents to bone is a promising strategy to reduce adverse effects and enhance efficacy at the desired site. Considering human bone is a tissue with unique properties due to its high content of HA, HA was chosen as a human bone model to quantitatively analyze the bone affinity of the platinum complexes. By using a high performance liquid chromatography (HPLC) method [22], the complex-HA binding assay was performed and the results showed that complexes **a** and **b** both have obvious affinity for HA (Figure 2). The adsorption quantity increased with the increase of the reaction time. When the reaction time is over 24 h, adsorption equilibrium can be achieved. The maximum adsorption percentages of complex **a** and complex **b** were 64% and 60%, respectively. This indicates that both complexes have moderate bone-targeting ability. As well known, bisphosphonates have higher affinities for the bone mineral, where the tridentate coordination of the P–C–P bone hook and R_1_ (OH) domains interact with the calcium site. The methyl esterification of hydroxyl groups on bisphosphonates may reduce the binding affinity to some extent.

### 2.4. Cytotoxicity

The cytotoxicity of complexes **a** and **b** against the human cancer cell lines HepG2, A549, U2OS, HCT116 and MDA-MB-231 was evaluated by the conventional MTT assay, in which the widely-used anticancer agent CDDP was used as the positive control. The antitumor activities were quantified by the corresponding IC_50_ values, as shown in Table 1. The results showed that both complexes exhibited inhibitory effects against the human cancer cell lines HepG2, A549, U2OS, HCT116 and MDA-MB-231, and the cell viability decreased with the increase of the drug concentration and the treatment time, especially on A549 and HepG2. The IC_50_ values of complex **a** against A549 after treated for 48 and 72 h were 180.23 and 108.72 μmol/L, respectively. The IC_50_ values of complex **a** against HepG2 after treated for of 48 and 72 h were 170.72 and 139.79 μmol/L, respectively. Complex **a** shows a slightly better cytotoxicity than complex **b**, but the cytotoxicities of **a** and **b** are both lower than that of CDDP. DNA is known to be the main target of platinum complexes, so the antitumor activity is related to the DNA-binding ability. The difference between the antitumor activity of platinum-dipicolyaminebisphosphonate ester complexes and CDDP may be attributed to their low DNA-binding ability and cancer cell uptake.

**Figure 3:**
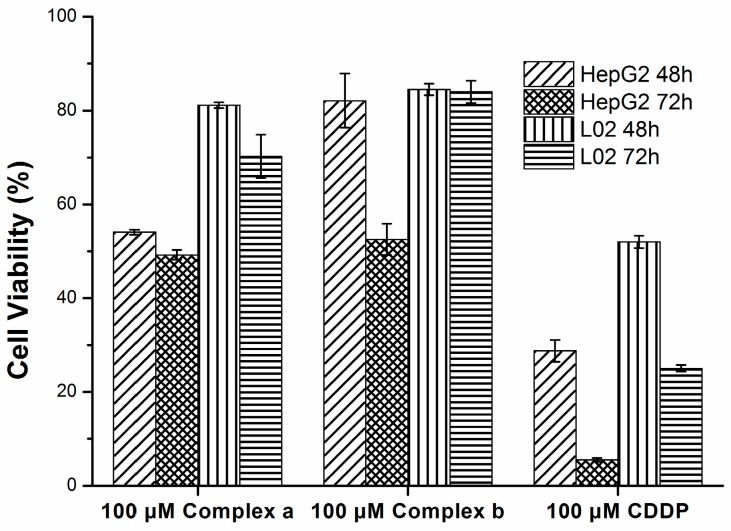
Inhibition effect of complexes **a**/**b** and CDDP on HepG2 and L02.

Hepatotoxicity usually limits the use of new drugs in the clinic. Therefore, the hepatotoxicity of two new complexes was evaluated against the L02 normal human liver cell line and compared with that of CDDP (Figure 3). The results showed that the cytotoxicity of both complexes against the normal human liver cell line L02 is far lower than that against the human liver carcinoma cell line HepG2. For instance, the cell viability of L02 treated by 100 μmol/L complex **a** for 72 h was 84.03%, while the cell viability of HepG2 was 52.54% after the same treatment. That is, the complex has a good selectivity for inhibiting human hepatocarcinoma cells rather than normal liver cells. In addition, the cell viability of L02 treated by a low concentration of CDDP (25 μmol/L) after 72 h was only 25.02%. This indicates that the hepatotoxicity of both complexes against the normal human liver cell lines L02 was lower than that of CDDP, although their anticancer efficacy is lower than that of CDDP.

### 2.5. Morphology Study

**Figure 4:**
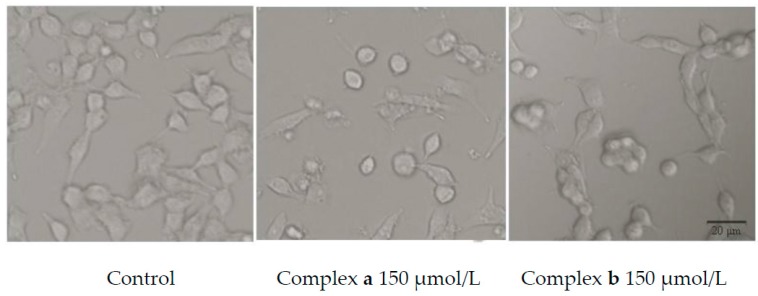
Effect of complexes **a**/**b** on the A549 cell morphology.

In order to investigate the effect of complexes **a** and **b** on tumor cell growth, the morphological changes of cancer cells were observed with an inverted microscope. As shown in Figure 4, after treatment with platinum complexes at the concentration of 150 μmol/L for 48 h, the density of cells decreased and morphology changed. For example, fragmentation, cell shrinkage and rounding can be observed. The results showed that two new platinum complexes can inhibit the growth and proliferation of cancer cells.

**Figure 5:**
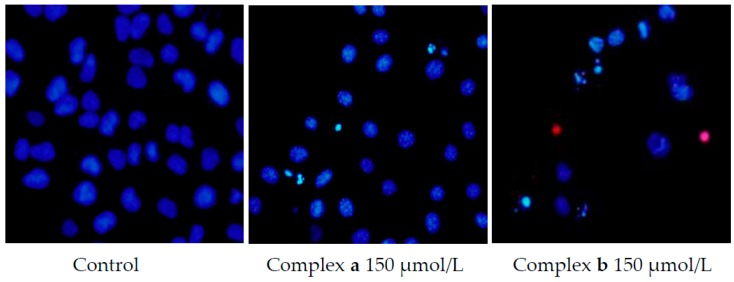
Apoptosis of A549 cell lines induced by complexes **a**/**b**.

To elucidate the mechanism of cell death, Hoechst 33342/propidium iodide double-staining experiments were carried out. As shown in Figure 5, after treatment with 150 μmol/L platinum complexes for 48 h, the nuclei of dead cells were penetrated with red fluorescence caused by propidium iodide, the living cells were stained by Hoechst 33342, and the control cells were stained with faint blue fluorescence. Brilliant blue color cell nucleus and apoptotic body can be observed in the drug group, which indicated that the new platinum complexes can induce cell apoptosis.

### 2.6. Cell Cycle Analysis

**Figure 6:**
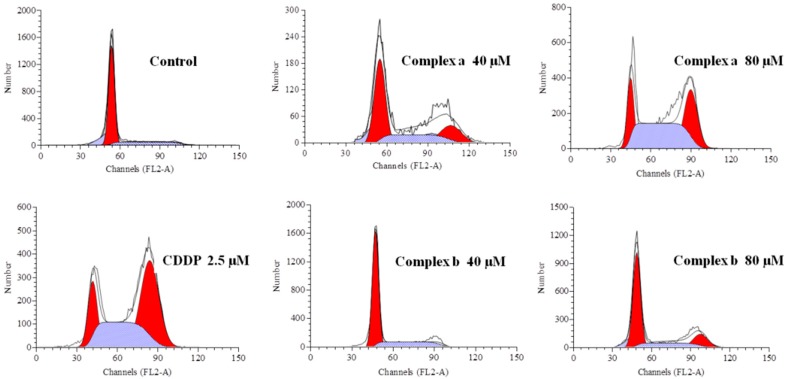
Propidium iodide (PI) assay of A549 cell lines treated by complexes **a**/**b** and CDDP.

The cell cycle distribution of cancer cell lines A549 induced by complexes **a** and **b** after 48 h treatment was investigated to study their anticancer mechanism of action. As shown in Figure 6 and Table 2, it is clear that after exposure to increasing concentrations of complexes **a** and **b**, the cell populations at the G0/G1 phase all decrease remarkably while those at the G2/M phase all increase. For example, the G0/G1 phase of A549 decreased from 75.28% of the control group to 17.89% of the drug group (80 μmol/L, complex **a**), while the G2/M phase of A549 increased from 2.37% of the control group to 44.95% of the drug group (80 μmol/L, complex **a**). This indicates that both complexes can cause the tumor cell cycle to arrest at the G2/M phase. It can also be deduced that the DNA damage caused by the two complexes will not be recovered at the M phase, which may be the reason for the cell apoptosis and death. Moreover, the flow cytometric detection results showed that these complexes can induce the cell cycle to arrest at the G2/M phase in a dose-dependent manner, thus exhibiting a dose-dependent inhibition effect on the cell proliferation. Investigating the cell cycle distribution treated by CDDP, a similar phenomenon was observed. Therefore, it is inferred that the anticancer action mechanism of these novel platinum complexes is similar to that of CDDP, which inhibits human cancer cell lines by inducing the cell cycle to arrest at the G2/M phase [13].

### 2.7. DNA Binding

**Figure 7:**
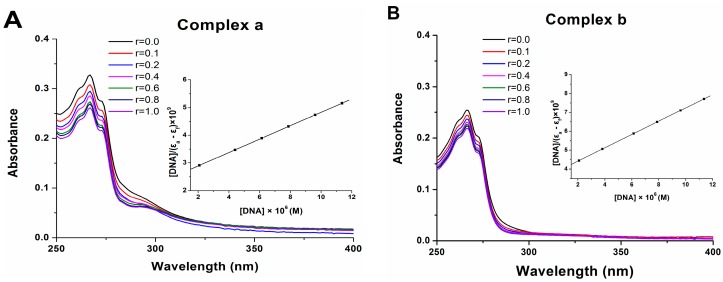
UV-Vis spectra of complex **a** (**A**) and **b** (**B**) upon addition of CT-DNA with the concentration ratio (r) of platinum complex to DNA of 0, 0.1, 0.2, 0.4, 0.6, 0.8 and 1.0, respectively (the insert is the plot of [DNA]/(ε_a_ − ε_f_) *vs.* [DNA]).

Electronic absorption spectroscopy and circular dichroism (CD) spectroscopy are usually utilized to determine the binding ability of complexes with the DNA helix. The intercalative interaction can be judged from the changes in the absorbance or intensity and the red shift in the wavelength. As seen from Figure 7, upon gradually addition of CT-DNA to a solution of complex **a** or **b**, a decrease in the absorption intensity (20% for complex **a** and 14% for complex **b**) accompanied with a negligible bathochromic shift (2 nm) was observed. The intercalation was characterized by hypochromism and red shift in the absorption spectroscopy for the intercalation involving stacking interaction [23]. The intercalating ability of complexes **a** and **b** with DNA was related to the planarity of the structure, and both platinum complexes interacted with CT-DNA in a partial intercalation mode. The binding constant (*K*_b_) of complexes **a** and **b** were 1.11 × 10^5^ M^−1^ and 9.48 × 10^4^ M^−1^, respectively. These results indicate that complexes **a** and **b** both have a moderate binding ability for DNA. The steric hindrance of platinum center coordinated by the chelating agent BPA maybe the reason for their moderate binding ability for DNA. This may also result in the weak cytotoxicity of these novel platinum complexes against human cancer cell lines.

**Figure 8:**
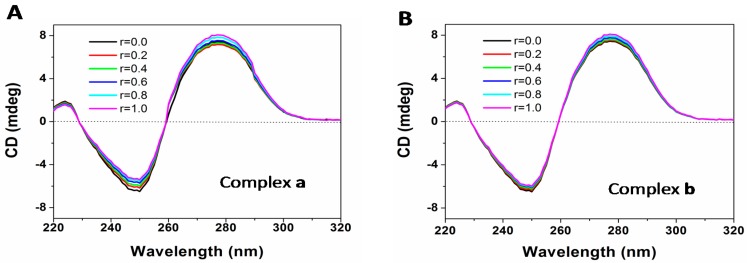
CD spectra of CT-DNA in the presence of increasing concentration of complex **a** (**A**) and **b** (**B**) with the concentration ratio (r) of platinum complex to DNA of 0, 0.2, 0.4, 0.6, 0.8, and 1.0, respectively.

CD spectroscopy appears to be the most effective tool for the analysis of the DNA-binding properties of various compounds. As can be seen from Figure 8, the characteristic CD spectrum of DNA exhibits a positive band around 275 nm due to the base stacking, a negative band around 245 nm due to the helicity of B-type DNA, and a crossover point near 258 nm, respectively. With the increasing concentration ratio (r) of platinum complex to CT-DNA, there is a slight intensity increase in the positive band located at 279 nm and a small intensity decrease in the negative band located at 250 nm, with a very small bathochromic shift. These changes are similar to those in the CD spectra of DNA induced by CDDP [24]. The enhancement of the positive band induced by addition of complex **a**/**b** indicates that the stacking of DNA base pairs increases, while the reduction in the negative band suggests that the stability of DNA double helix structure decreases. The increase in the ellipticity of the positive band might be attributed to the additional stabilization of base stacking derived from the covalent binding between the metal center Pt and guanine or cytosine groups and other noncovalent interactions upon DNA-platinum complex formation, while the slight decrease in the ellipticity of the negative band suggested that complexes **a**/**b** could only slightly unwind the DNA helix. It is reasonable to hypothesize that the occurrence of small DNA conformational distortions was induced by the monodentate binding interaction between the Pt(II)-complex and the nucleobase [25]. Furthermore, the Pt(II) center coordinated by three N atoms from BPA will not cause any major conformational distortion after the release of the leaving group.

## 3. Materials and Methods

### 3.1. General Information

All chemicals were purchased as reagent grade and used without further purification. *cis*-Pt(DMSO)_2_Cl_2_ was synthesized according to a previous literature report [18]. Electrospray ionization–mass spectrometry (ESI-MS) was performed using a Waters Platform ZMD4000 liquid chromatography–mass spectrometry (LC-MS) (Waters Corporation, Milford, MA, USA). Nuclear magnetic resonance (NMR) spectra were obtained with a Bruker DRX-500 spectrometer (Bruker Corporation, Karlsruhe, Germany), and the chemical shift values were referenced to the internal standard tetramethylsilane (TMS). Elemental analyses were performed with an Elementar VARIO EL III Elemental Analyzer (Perkin Elmer Corporation, Waltham, MA, USA). IR spectra were recorded in the range of 400–4000 cm^−1^ with a Bruker Vector 22 FT-IR spectrophotometer (Bruker, Fremont, CA, USA). HPLC analyses were carried out using a Waters 600 system (Waters Corporation) equipped with a UV detector. The CD spectra were recorded with a MOS 450 spectropolarimeter (BioLogic, Claix, France), and the UV-Vis spectra were recorded with a Lamda 25 spectrometer (Perkin-Elmer Corporation, Waltham, MA, USA). 

The human cancer cell lines U2OS (osteosarcoma), A549 (lung cancer), HCT116 (colon carcinoma), MDAMB-231 (breast cancer) and HepG2 (liver carcinoma) as well as the human normal liver cell line L02 were obtained from the Cell Bank of Chinese Academy of Sciences (Shanghai, China). The reagent 3-[4,5-dimethyl-2-thiazolyl]-2,5-diphenyl-2-tetrazolium bromide (MTT) used for cell lysis was purchased from Sigma (Saint Louis, MO, USA). Dulbecco’s modified Eagle medium (DMEM) and Roswell Park Memorial Institute (RPMI-1640) medium were purchased from Gibco Company (Gaithersburg, MD, USA). Dimethyl sulfoxide (DMSO), propidium iodide (PI), RNase inhibitor and Hoechst 33342 were purchased from Beyotime Institute of Biotechnology (Haimen, China). Cell culture plates were products of Corning (Acton, MA, USA). Calf thymus DNA (CT-DNA) was purchased from Sigma-Aldrich and used as received.

### 3.2. Preparation of Ligands and Complexes

#### 3.2.1. Preparation of Ligands

The dipycolylaminepamidronate and dipycolylamine alendronate were synthesized according to the published methods [16].

*Dipycolylaminepamidronate ester.* An aliquot of trimethylsilyldiazomethane (1.0 mL) was added to dipycolylaminepamidronate (50 mg, 120 μmol) in methanol (2 mL) under nitrogen in the dark. The samples were evaporated to dryness and purified by flash column chromatography on silica gel to give dipycolylaminepamidronate (26 mg, 47%) as the yellow oil. ^1^H-NMR (400 MHz, CD_3_OD): δ_H_ (ppm); 8.403 (2H, d, *J* = 3.81 Hz, PyH), 7.776 (2H, t, *J* = 7.47 Hz, PyH), 7.411 (2H, d, *J* = 7.60 Hz, PyH), 7.254 (2H, t, *J* = 6.15 Hz, PyH), 4.203 (4H, s, Py-CH_2_-N), 3.632 (6H, s, CH_3_), 3.595 (6H, s, CH_3_), 3.206 (2H, t, *J* = 7.04 Hz, N-CH_2_), 2.258–2.374 (2H, m, CH_2_). ^13^C-NMR (100.6 MHz, CD_3_OD): δ_C_ (ppm); 157.173 (2C, Py), 148.051 (2CH, Py), 137.346 (2CH, Py), 128.701 (2CH, Py), 126.747 (2CH, Py), 70.247 (t, *J_C-P_* = 127.66 Hz, CH_2_-C(PO3)_2_(OH)), 59.282 (C, PyCH_2_), 55.896 (NCH_2_), 53.723(CH_3_), 31.392 (CH_2-_C(PO3)_2_(OH)). ^31^P NMR (161.9 MHz, CD_3_OD): δp (ppm); 21.429. ESI-MS (*m*/*z*): 474.5 for [M + H]^+^. IR (KBr)/cm^−1^: 3440 (s), 1641 (m), 1247 (s), 1025 (s). Elemental analysis (%): calcd. for C_19_H_29_N_3_P_2_O_7_: C, 48.10; H, 4.01; N, 8.86. found: C, 47.93; H, 4.07; N, 8.83.

*Dipycolylaminealendronate ester.* An aliquot of trimethylsilyldiazomethane (1.0 mL) was added to dipycolylaminealendronate (50 mg, 116 umol) in methanol (2 mL) under nitrogen in the dark. The method was the same as that described above and a yellow oil was obtained (23.1 mg, 50%). ^1^H-NMR (400 MHz, CD_3_OD): δ_H_ (ppm); 8.265 (2H, d, *J* = 4.03 Hz, PyH), 7.768 (2H, t, *J* = 7.80 Hz, PyH), 7.425 (2H, d, *J* = 4.89 Hz, PyH), 7.344 (2H, t, *J* = 6.42 Hz, PyH), 3.831 (4H, s, Py-CH_2_-N), 3.652 (6H, s, CH_3_), 3.634 (6H, s, CH_3_), 3.045 (2H, t, *J* = 7.81 Hz, N-CH_2_), 2.254–2.294 (2H, m, CH_2_), 2.142–2.212 (2H, m, CH_2_). ^13^C-NMR (100.6 MHz, CD_3_OD): δ_C_ (ppm); 157.084 (2C, Py), 148.280 (2CH, Py), 137.341 (2CH, Py), 128.814 (2CH, Py), 126.836 (2CH, Py), 69.408 (t, *J_C-P_* = 145.67 Hz, C(PO3)_2_(OH)), 58.993 (C, PyCH_2_), 55.803 (NCH_2_), 53.107 (CH_3_), 30.567 (CH_2_-C(PO_3_)_2_(OH)), 20.136 (CH_2_). ^31^P NMR (161.9 MHz, CD_3_OD): δp (ppm); 21.209. ESI-MS (*m*/*z*): 488.6 for [M + H]^+^. IR (KBr)/cm^−1^: 3430 (s), 1630 (m), 1210 (s), 1040 (s). Elemental analysis (%): calcd. for C_20_H_31_N_3_P_2_O_7_: C, 49.18; H, 6.35; N, 8.60. found: C, 49.23; H, 6.28; N, 8.63.

#### 3.2.2. Preparation of Platinum Complexes

*[Pt(DPE)Cl]Cl* (**a**). A solution of *cis*-Pt(DMSO)_2_Cl_2_ (46 mg, 0.11 mmol) was added to dipycolylaminepamidronate ester (50 mg, 0.11 mmol) dissolved in anhydrous methanol (2 mL). The mixture was stirred in the dark at room temperature for 24 h. The samples were purified by flash column chromatography on silica gel. Yield: 71%. ^1^H-NMR (400 MHz, CD_3_OD): δ_H_ (ppm); 8.613 (2H, d, *J* = 4.50 Hz, PyH), 7.819 (2H, td, *J* = 7.65, 1.28 Hz; PyH), 7.528 (2H, d, *J* = 7.94 Hz, PyH), 7.495 (2H, t, *J* = 6.85 Hz, PyH), 4.356 (4H, s, Py-CH_2_-N), 3.605 (6H, s, CH_3_), 3.567 (6H, s, CH_3_), 3.079 (2H, t, *J* = 7.26 Hz, N-CH_2_), 2.132–2.197 (2H, m, CH_2_). ^13^C-NMR (100.6 MHz, CD_3_OD): δ_C_ (ppm); 158.848 (2C, Py), 149.902 (2CH, Py), 137.824 (2CH, Py), 129.081 (2CH, Py), 127.121 (2CH, Py), 68.753 (t, *J*_C-P_ = 127.66 Hz, C(PO_3_)_2_(OH)), 60.821 (C, PyCH_2_), 55.806 (NCH_2_), 53.580 (CH_3_), 30.619 (CH_2_). ^31^P NMR (161.9 MHz, CD_3_OD): δp (ppm); 20.436. ESI-MS (*m*/*z*): 704.1 for [M − Cl]^+^. IR (KBr)/cm^−1^: 3434 (m), 1612 (s), 1257 (s), 1027 (s). Elemental analysis (%): calcd. for C_19_H_29_N_3_P_2_O_7_PtCl_2_: C, 30.85; H, 3.92; N, 5.68. found: C, 30.81; H, 3.95; N, 5.71.

*[Pt(DAE)Cl]Cl* (**b**). The same procedure was performed for the preparation of complex **b** only dipycolylaminealendronate ester was used instead of dipycolylaminepamidronate ester. Yield: 66%. ^1^H-NMR (400 MHz, CD_3_OD): δ_H_ (ppm); 8.508 (2H, d, *J* = 4.40 Hz, PyH), 7.838 (2H, td, *J* = 7.89, 1.65 Hz; PyH), 7.552 (2H, d, *J* = 7.98 Hz, PyH), 7.468 (2H, t, *J* = 6.24 Hz, PyH), 3.970 (4H, s, Py-CH_2_-N), 3.694 (6H, s, CH3), 3.662 (6H, s, CH3), 3.076 (2H, t, *J* = 6.40 Hz, N-CH_2_), 2.336 (2H, m, CH_2_), 2.046–2.104 (2H, m, CH_2_). ^13^C-NMR (100.6 MHz, CD_3_OD): δ_C_ (ppm); 158.859 (2C, Py), 149.918 (2CH, Py), 137.779 (2CH, Py), 129.316 (2CH, Py), 127.408 (2CH, Py), 66.307 (t, *J*_C-P_ = 133.99 Hz, C(PO_3_)_2_(OH)), 60.596 (C, PyCH_2_), 55.894 (NCH_2_), 53.405 (CH_3_), 31.026(CH_2_), 20.943 (CH_2_). ^31^P NMR (161.9 MHz, CD_3_OD): δp (ppm); 20.245. ESI-MS (*m*/*z*): 718.3 for [M − Cl]^+^. IR (KBr)/cm^−1^: 3400 (s), 1594 (m), 1251 (s), 1027 (s). Elemental analysis (%): calcd. for C_20_H_31_N_3_P_2_O_7_PtCl_2_: C, 31.87; H, 4.11; N, 5.57. found: C, 31.82; H, 4.12; N, 5.59.

### 3.3. Lipid-Water Partition Coefficient

The UV-Vis absorbance of the aqueous phase was measured at 267 nm and the concentrations of platinum complexes were determined by standard curves, which were plotted from the ultraviolet absorption of different concentrations (Figure S1 of Supplementary Materials). *n*-Octanol and aqueous partition coefficients were measured at room temperature. Saturated *n*-octanol (2 mL) was added to the platinum complex solution (50 mg/L, 2 mL) and mixed in a vortex apparatus. After 24 h, the mixture solution was centrifuged at 2000 rpm for 5 min and the concentration of platinum complexes was determined by UV-Vis spectroscopy. The partition coefficient can be calculated from the ratio of the content between the *n*-octanol and the aqueous phase using the following formula:

(1) $$\text{logP}=\text{log}C_{n-\text{octanol}}/C_{water}$$

### 3.4. Bone Binding Assay

The HA binding assay was set up to investigate the bone targeting ability of two platinum complexes using a HPLC method. The HPLC system was equipped with a Waters 1525 binary pump and a Waters 2487 dual wavelength absorbance detector, and a reverse phase C_18_ column was used for HPLC analysis. The flow rate was 1.0 mL/min. The concentrations of platinum complexes were determined by standard curve, which was plotted from the integral area of different concentrations (Figure S2 of Supplementary Materials). HA (10 mg) was added to different concentrations of the platinum complex solution (2 mL) and incubated at 37 °C in a vortex apparatus for 48 h. Then the mixture solution was centrifuged at 2000 rpm for 5 min and filtered through microporous filter. The complex remained in the solution was quantified using HPLC method. The rate of the complex adsorbed onto HA was determined according to the following formula:

(2) $$A\%=\frac{m-m_{0}}{m}\times 100\%$$

where *m* is the initial amount of platinum complex and *m_0_* is the remaining amount of platinum complex.

### 3.5. Cell Culture and Drug Treatment

Human osteosarcoma cell lines (U2OS), breast cancer cell lines (MDA-MB-231), colon cancer cell lines (HCT-116), hepatoma cell lines (HepG2), and normal hepatic cell lines (L02) were cultured in DMEM (Gibco) with 10% heat-inactivated fetal bovine serum, 100 U/mL penicillin and 100 U/mL streptomycin. Human lung adenocarcinoma cells (A549) were maintained in RPMI-1640 medium (Gibco) with 10% heat-inactivated fetal bovine serum, 100 U/mL penicillin and 100 U/mL streptomycin. These cells were cultured at 37 °C in an atmosphere of 5% CO_2_ and 100% relative humidity.

### 3.6. Cytotoxicity Assay

The cytotoxicity of platinum complexes was measured by the conventional MTT [3-(4,5-dimethylthiazol-2-yl)-2,5-diphenyltetrazolium bromide] assay in 96-well plates at a cell density of 7 × 10^3^ cells/mL. After cell inoculation, the plates were incubated overnight. Then the platinum compounds were added to the culture medium to give the indicated final concentration, and cells were then incubated for additional 48 or 72 h. The medium was removed and 20 μL MTT (5 mg/mL, PBS) was added to each well and incubated for additional 4 h. Then, 150 μL DMSO was added to each well and the plate was vibrated to lyse the precipitate. The absorbance of each well was measured at 490 nm with a Bio-Rad Model 3550-UV microplate reader (Bio-Rad Laboratories, Hercules, CA, USA) to calculate the inhibition rate and IC_50_.

### 3.7. Morphology Study

The A549 cells were seeded in 96-well plates at a density of 7 × 10^3^ cells/mL in 100 μL culture medium and incubated overnight. Then the platinum complexes were added to each well at a concentration of 150 μmol/L. After incubation for 48 h, the cell morphology changes were photographed by the inverted light microscopy with a Lumix DMC-FH2 instrument (Panasonic, Osaka, Japan).

The A549 cells were seeded in 96-well plates at a density of 7 × 10^3^ cells/mL in 100 μL culture medium and incubated overnight. Then the platinum complexes were added to the plates at a concentration of 150 μmol/L. After incubation for 48 h, the A549 cells were incubated with 10 μg/mL Hoechst 33342 for 15 min and 10 μg/mL PI for an additional 15 min, and then washed with phosphate buffered saline (PBS). The cells were observed and photographed by inverted fluorescence microscopy.

### 3.8. Cell Cycle Analysis

The A549 cells were seeded at a density of 2 × 10^6^ cells/mL in 6-well plates and incubated overnight. Then the platinum complexes were added to the plates at a concentration of 40 and 80 μmol/L, respectively. After incubation for 48 h, cells were harvested, washed twice with PBS, suspended with 70% ethanol (stored at −20 °C) and then fixed at 4 °C overnight. The cells were centrifuged to remove ethanol, washed with PBS, and then resuspended in 1 mL of DNA staining reagent containing 50 mg/mL RNase, 50 mg/mL PI, 0.1% Triton X-100 and 0.1 mM EDTA (pH 7.4) for 30 min at 4 °C. Cells were kept shielded from light before being analyzed by flow cytometry. Finally, the cell cycle profile in each sample was analyzed using a FACS Calibur flow cytometer (Becton Dickinson, San Jose, CA, USA) equipped with a 488 nm argon ion laser and the Cell Quest 3.1 software. DNA histograms obtained from Cell Quest 3.1 were further analyzed under the Windows operating system environment using ModFit LT 3.2.1 (Verity Software House, Topsham, ME, USA).

### 3.9. DNA Binding Assay

CT-DNA was dissolved in the buffer solution (5 mM Tris–HCl, 50 mM NaCl, pH 7.4) as a stock solution, which was stored at 4 °C and used within 3 days. The solution gives a UV absorbance ratio (A_260_/A_280_) of *ca*. 1.8, indicating that CT-DNA was sufficiently protein-free. The concentration of CT-DNA was determined by measuring the UV absorption at 260 nm, taking 6600 M^−1^/cm as its molar absorption coefficient.

The UV-Vis spectra titration used Tris-HCl buffer as the reference solution. The CT-DNA (2 μL) was gradually added to the solution of platinum complex in Tris-HCl buffer and the absorption spectrum was recorded until no obvious change observed in the spectrum. The DNA binding constant can be determined by the following equation [26]:

(3) $$[\text{DNA}]/(\varepsilon_{a}-\varepsilon_{f})=[\text{DNA}]/(\varepsilon_{b}-\varepsilon_{f})+1/K_{b}\left(\varepsilon_{b}-\varepsilon_{f}\right)$$

where ε_a_, ε_f_ and ε_b_ correspond to the apparent extinction coefficient (Absorb/[platinum complexes]), the extinction coefficient for the free platinum complex and the extinction coefficient for the platinum complex in the fully bound form, respectively. [DNA] represents the concentration of CT-DNA. A plot of [DNA]/(ε_a_ − ε_f_) *vs.* [DNA] gives the binding constant *K*_b_ as the ratio of the slope to the intercept.

The stock solutions of complexes **a** and **b** used for the CD experiments were prepared using Tris-HCl buffer (5 mM Tris-HCl, 50 mM NaCl, pH 7.4). The CD spectra for CT-DNA were recorded as follows: each sample of CT-DNA (1.0 × 10^−4^ M) was incubated with different concentrations of complex **a** or **b** (concentration ratio of platinum complex to DNA is 0, 0.1, 0.2, 0.4, 0.6, 0.8 and 1.0, respectively) for 24 h in the dark and scanned in the wavelength range of 220–320 nm at a speed of 10 nm/min, where the buffer background was subtracted.

## 4. Conclusions

In summary, two novel platinum complexes bearing bisphosphonate esters were designed and synthesized. Both complexes exhibited targeting affinity for the bone mineral hydroxyapatite, and the maximum adsorption percentage was 64% for complex **a** and 60% for complex **b**, respectively. The two platinum complexes have good hydrophilicity, which is beneficial for increasing their bone-targeting ability. They both showed cytotoxic effects on human cancer cell lines and low cytotoxicity against the human normal liver cell line L02. The complexes inhibited the growth and proliferation of human cancer cell lines by inducing cell apoptosis and cell arrest at the G2/M phase. The new complexes have a moderate DNA affinity and interact with DNA in a partial intercalation mode, which leads the helical structure of DNA to become looser and the base stacking interactions to become stronger. This work may provide a reference for the design and synthesis of novel bone-targeting antitumor complexes. Further work for developing novel platinum-based complexes with higher anticancer activity and lower side effects are underway.

## Figures and Tables

**Table 1:** IC_50_ values of complexes **a**/**b** and CDDP against five human cancer cell lines.

Complex	IC_50_ (μmol/L) ± SD				
	U2OS	A549	HCT116	MDA-MB-231	HepG2
CDDP (48 h)	11.04 ± 0.34	19.85 ± 1.49	16.20 ± 0.44	29.94 ± 1.65	6.78 ± 0.54
CDDP (72 h)	10.82 ± 0.37	15.38 ± 0.58	11.80 ± 0.26	14.05 ± 1.01	5.63 ± 0.34
Complex **a** (48 h)	251.74 ± 1.97	180.23 ± 0.93	273.02 ± 1.41	190.59 ± 0.34	170.72 ± 0.66
Complex **a** (72 h)	166.41 ± 1.04	108.72 ± 5.34	209.50 ± 0.80	111.13 ± 1.37	139.79 ± 0.42
Complex **b** (48 h)	262.92 ± 6.46	258.17 ± 3.88	638.47 ± 9.65	324.54 ± 5.83	271.12 ± 1.80
Complex **b** (72 h)	202.82 ± 0.94	237.31 ± 0.39	376.59 ± 0.43	245.15 ± 6.68	160.06 ± 3.20

**Table 2:** Cell cycle distribution of A549 cell lines induced by complexes **a**/**b** and CDDP.

Phase	Control	Complex a	Complex a	Complex b	Complex b	CDDP
		40 μM	80 μM	40 μM	80 μM	2.5 μM
G0/G1 (%)	75.28	58.79	17.89	73.30	45.78	17.49
S (%)	22.35	16.17	37.16	18.59	24.58	43.06
G2/M (%)	2.37	25.04	44.95	7.71	29.66	39.45

## Supplementary Materials

Supplementary materials can be accessed at: http://www.mdpi.com/1420-3049/21/3/255/s1.
