# Supplementary material for: Preparation and Biological Evaluation of Two Novel Platinum(II) Complexes Based on the Ligands of Dipicolyamine Bisphosphonate Esters
Source: Molecules. 2016 Feb 24;21(3):255. doi: 10.3390/molecules21030255 (PMC6272982; doi:10.3390/molecules21030255)
Supplement: Supplementary file 1 [file molecules-21-00255-s001.pdf]

Supplementary Materials: Preparation and Biological Evaluation of Two Novel Platinum(II) Complexes Based on the Ligands of Dipicolyamine Bisphosphonate Esters

Ling Qiu, Hong Liu, Ke Li, Gaochao Lv, Hui Yang, Xiaofeng Qin and Jianguo Lin

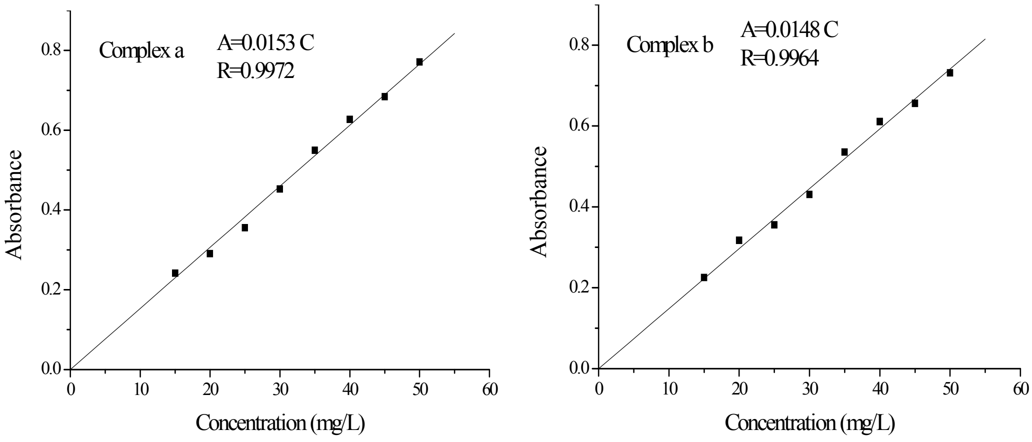

Figure S1. Standard curves of complexes **a** and **b** determined by UV-Vis absorption spectra in measuring the lipid-water partition coefficient of complex.

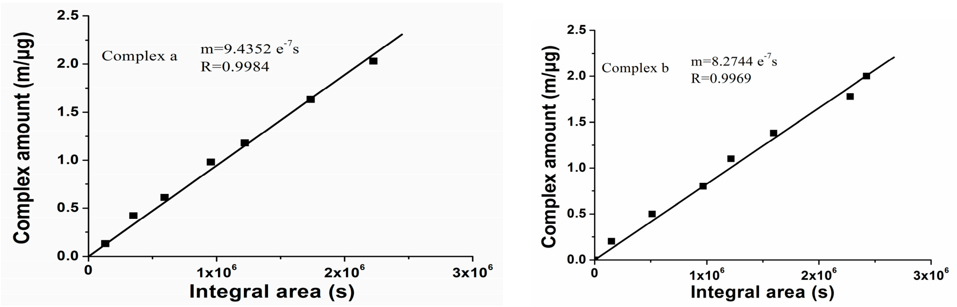

Figure S2. Standard curves of complexes **a** and **b** used in the HA-binding assay.

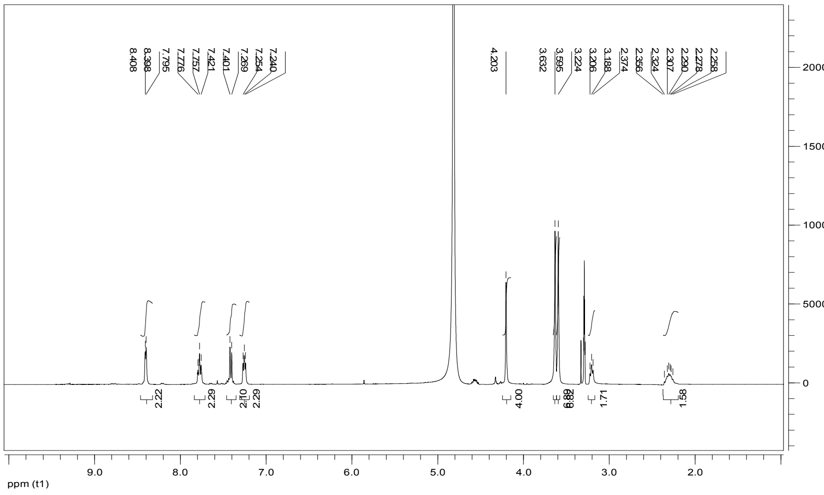

Figure S3. ^{1}H-NMR of ligand DPA (CD_3OD).

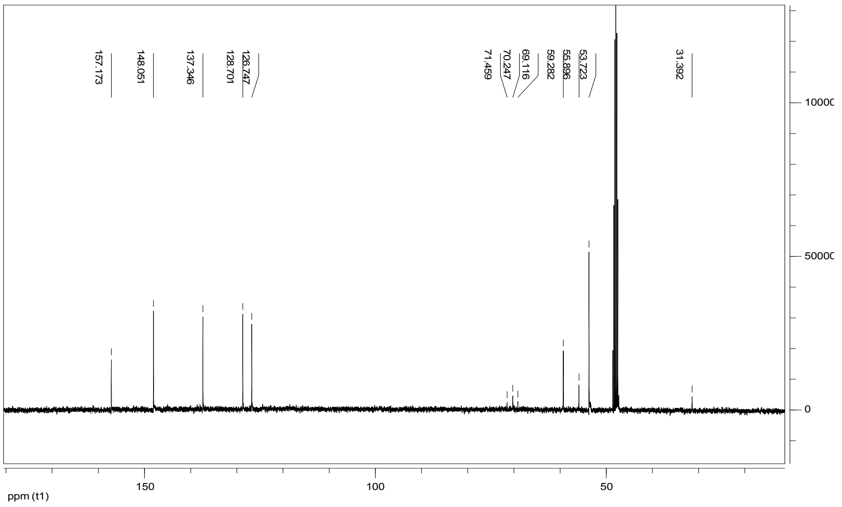

Figure S4. ^{13}C-NMR of ligand DPE (CD_3OD).

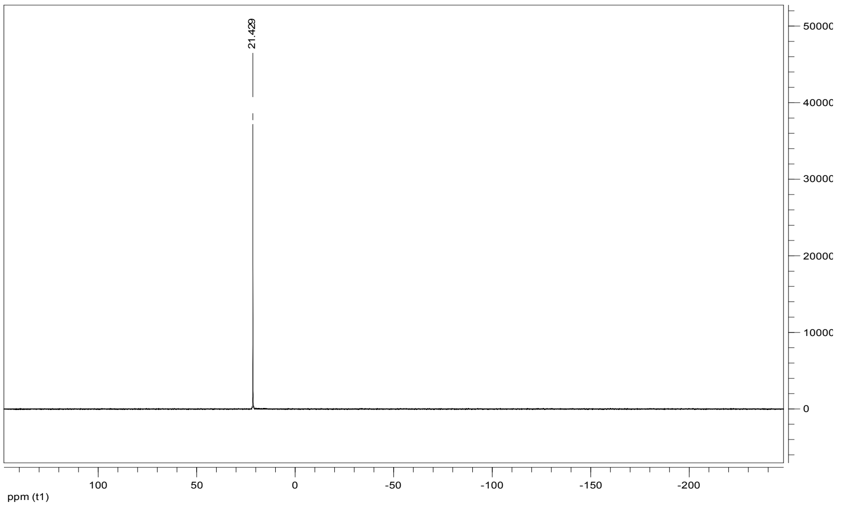

Figure S5. ^{31}P-NMR of ligand DPE (CD_3OD).

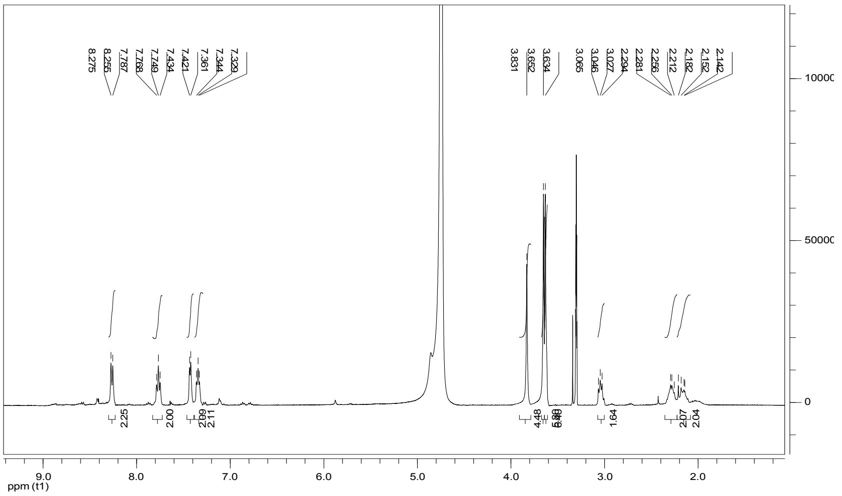

Figure S6. ^{1}H-NMR of ligand DAE (CD_3OD).

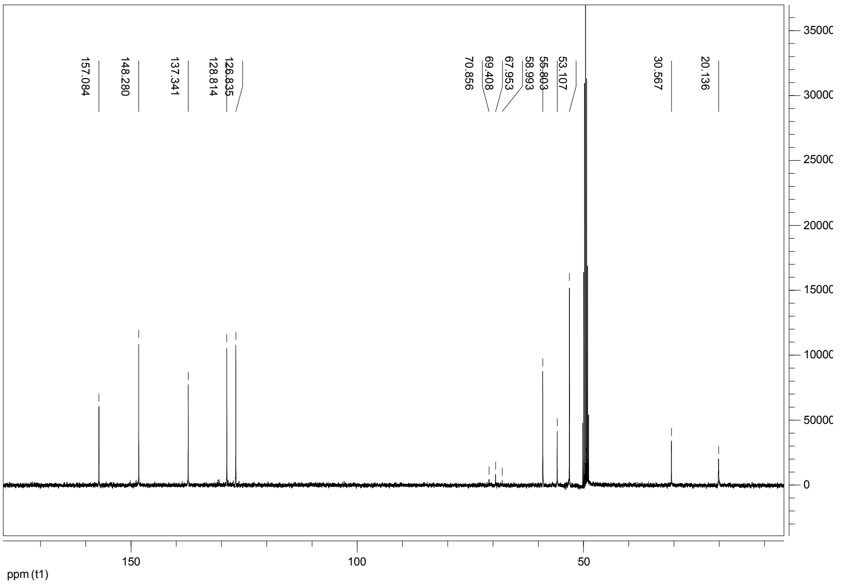

Figure S7. ^{13}C-NMR of ligand DAE (CD_3OD).

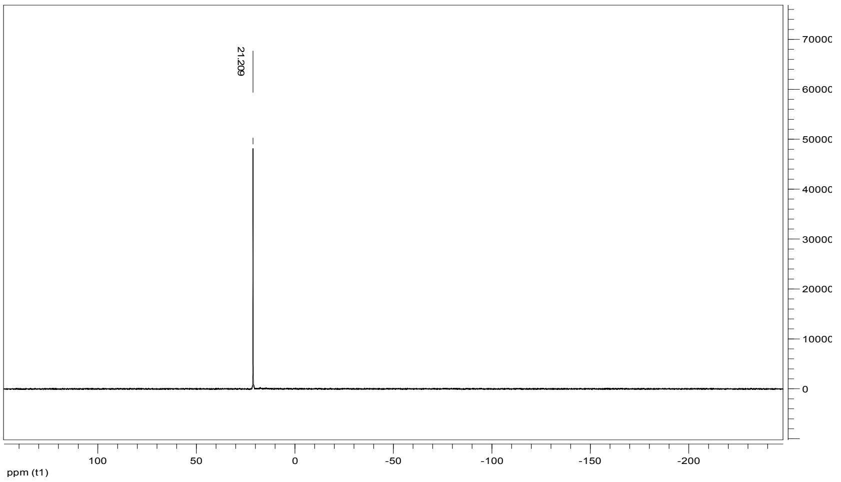

Figure S8. ^{31}P-NMR of ligand DAE (CD_3OD).

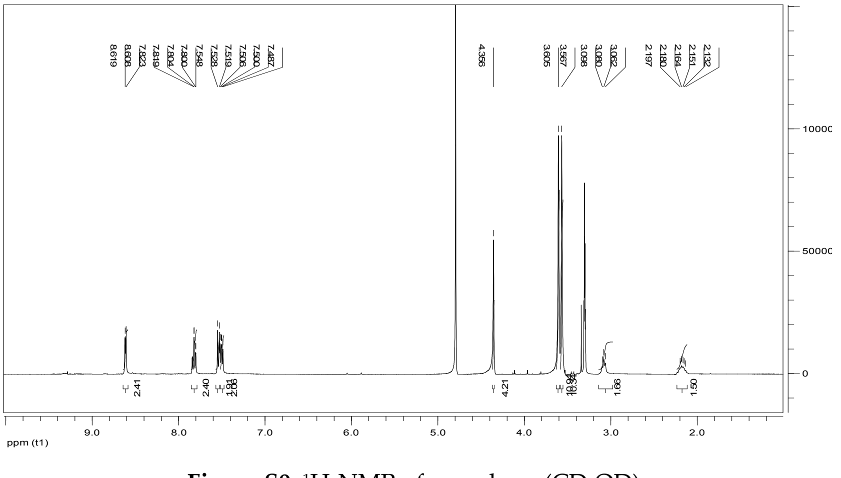

Figure S9. ^{1}H-NMR of complex **a** (CD_3OD).

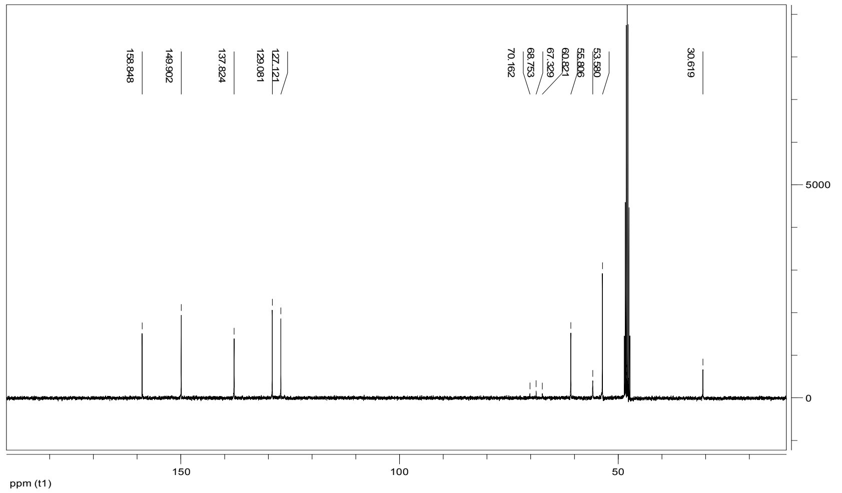

Figure S10. ^{13}C-NMR of complex **a** (CD_3OD).

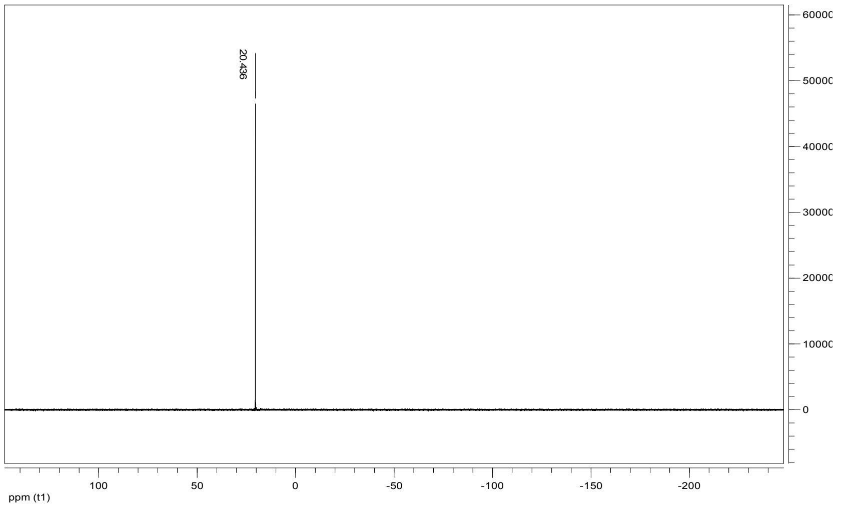

Figure S11. ^{31}P-NMR of complex **a** (CD_3OD).

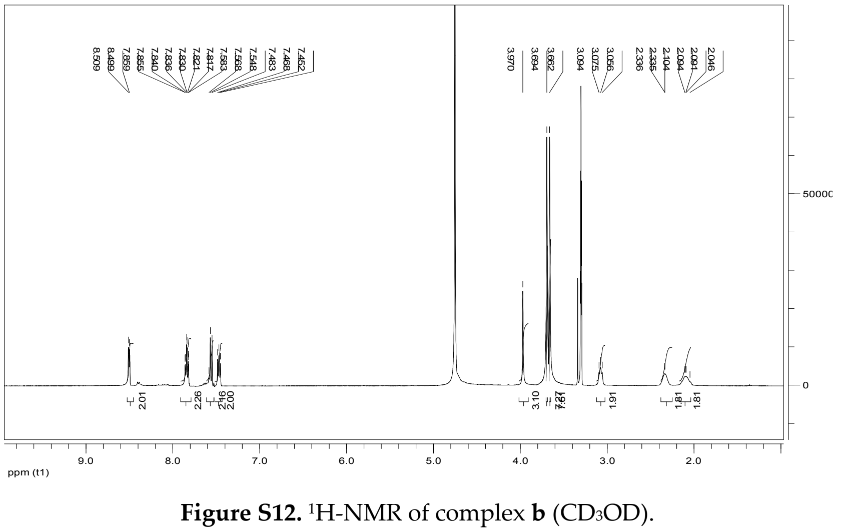

Figure S12. ^{1}H-NMR of complex **b** (CD_3OD).

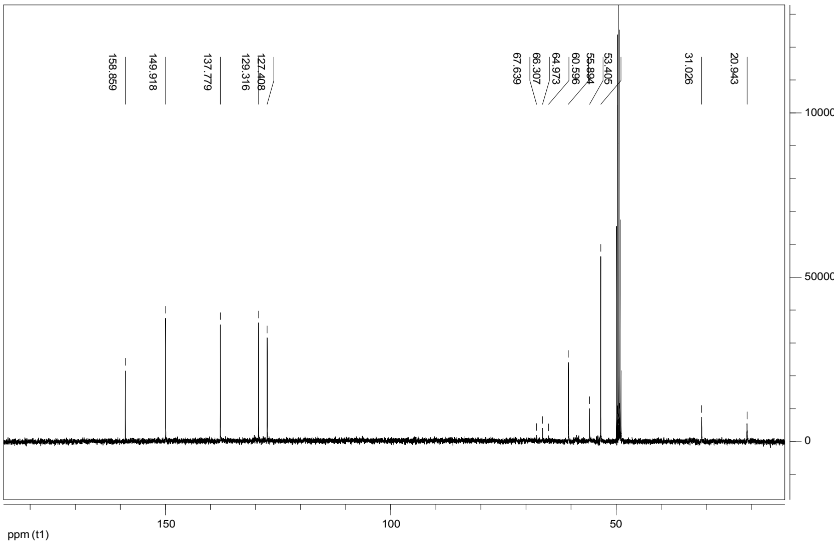

Figure S13. ^{13}C-NMR of complex **b** (CD_3OD).

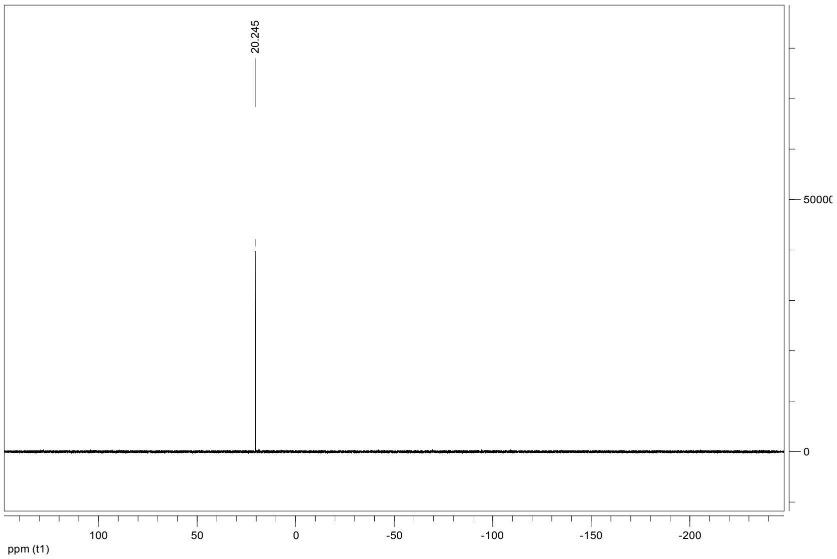

Figure S14. ^{31}P-NMR of complex **b** (CD_3OD).
